# Clinical Applications of “In-Hospital” 3D Printing in Hip Surgery: A Systematic Narrative Review

**DOI:** 10.3390/jcm13020599

**Published:** 2024-01-20

**Authors:** Ignacio Aguado-Maestro, Clarisa Simón-Pérez, Manuel García-Alonso, Juan José Ailagas-De Las Heras, Elena Paredes-Herrero

**Affiliations:** 1 Department of Traumatology and Orthopedic Surgery, Río Hortega University Hospital, 47012 Valladolid, Spain; 2 Institute of Orthopedic Surgery and Traumatology (ICOTVA), Hospital Sagrado Corazón, 47002 Valladolid, Spain; 3 Department of Traumatology and Orthopedic Surgery, Clínico University Hospital, 47003 Valladolid, Spain; 4 Department of Neurosurgery, Clínico University Hospital, 47003 Valladolid, Spain

**Keywords:** 3D printing, hip surgery, PSI

## Abstract

***Introduction***: Interest in 3D printing for orthopedic surgery has been increasing since its progressive adoption in most of the hospitals around the world. The aim of the study is to describe all the current applications of 3D printing in patients undergoing hip surgery of any type at the present time. ***Materials and Methods***: We conducted a systematic narrative review of publications indexed in MedLine through the search engine PubMed, with the following parameters: 3D printing AND (orthopedics OR traumatology) NOT tissue engineering NOT scaffold NOT in vitro and deadline 31 July 2023. After reading the abstracts of the articles, papers were selected according to the following criteria: full text in English or Spanish and content related to hip surgery. Those publications involving experimental studies (in vitro or with anatomical specimens) or 3D printing outside of hospital facilities as well as 3D-printed commercial implants were excluded. Results are presented as a reference guide classified by disease, including the used software and the steps required for the development of the idea. ***Results***: We found a total of 27 indications for in-house 3D printing for hip surgery, which are described in the article. ***Conclusions***: There are many surgical applications of 3D printing in hip surgery, most of them based on CT images. Most of the publications lack evidence, and further randomized studies should be encouraged to assess the advantages of these indications.

## 1. Introduction

Three-dimensional printing, also known as additive manufacturing, is the process of creating objects from a 3D digital model layer by layer. Its origin dates back to 1984, when Chuck Hall developed his patent “Apparatus for production of three-dimensional objects by stereolithography” [1]. One of the fundamental advantages of 3D printing, apart from its ability to print objects of great geometric complexity, is that the cost of printing is not affected by the complexity of the object. The patent’s liberation and the drop in printer prices have allowed orthopedic surgeons to adopt this type of technology. Its limitless possibilities have made the boundaries of applications practically dependent on the imagination of the healthcare professionals involved. A full scheme of the 3D printing workflow can be found under Appendix A.

Current applications of in-hospital 3D printing are overwhelmingly increasing every year and can be summarized in three groups: (1) preoperative planning (which includes fracture and bone defect comprehension, pre-surgical plate bending, as well as applications in oncologic surgery) [2,3,4]; (2) manufacturing of patient-specific surgical guides or surgical tools [5,6]; and (3) teaching and learning of students, residents or specialists [7,8,9,10,11]. Many publications have arisen involving applications of this technology in orthopedic surgery. It is important to note that at the present time, most of the publications intend to show the possibilities of the technology (with very small samples, single-patient studies occasionally), and there is still a lack of clinical trials that prove the benefits of these techniques with regards to the traditional methods. Most of the reviews published at the present time are not systematic and involve general orthopedic applications [12,13,14,15,16,17]. Certain hip diseases, such as acetabular fractures and revision total hip arthroplasty, benefit from numerous publications and systematic reviews [18,19,20,21]; however, there are no systematic reviews at the present time in which all current applications of 3D printing in hip orthopedic surgery are summarized. The aim of this study is to conduct the first systematic review of all current in-house 3D printing applications in orthopedic and trauma surgery of the hip. The intent is that this review will serve as a comprehensive reference guide for surgeons specializing in hip procedures.

## 2. Materials and Methods

We conducted a systematic search and narrative review of publications from MEDLINE via the National Library of Medicine’s PubMed search engine. We decided to choose PubMed as the database for this study because it is specialized in biomedical literature, being the preferred choice for topics related to surgery and orthopedics. It also has advanced search features (MeSH) and covers many prestigious and specialized peer review publications in orthopedics, increasing the likelihood of finding relevant and high-quality articles. The review was not registered in the PROSPERO system as it does not accept registrations for this type of literature reviews. The PRISMA method was used to conduct the scheme of the publication (Appendix B). All studies employing 3D printing in orthopedic and trauma surgery through in-house design and/or printing within hospital facilities with surgical applications involving the hip were collected. The last search was conducted on 31 August 2023. Articles published until 31 July 2023 were included.

The search terms used in PubMed were: 3D printing AND (orthopedics OR traumatology) NOT tissue engineering NOT scaffold NOT in vitro.

After reading the abstracts of the articles, the following inclusion criteria were applied: articles in English or Spanish, availability of full-text articles, reference to 3D printing applications in orthopedic and trauma surgery of the hip. Exclusion criteria included: articles published in languages other than English or Spanish; inability to access the full text of the article; articles related to animal or veterinary experimentation; articles unrelated to orthopedic or trauma surgery; experimental studies involving non-human subjects or specimens; studies involving cellular therapy or tissue engineering; articles related to the use of custom prostheses or implants, industrial manufacturing, orthoses or navigation; articles based on 3D printing workflows developed entirely outside of hospital facilities.

After selecting the articles, all of them were downloaded in full-text PDF format and included in a Zotero database (Zotero 6 for Mac, version 6.0.15, Corporation for Digital Scholarship, Vienna, VA, USA). They were classified based on their content into the following categories: preoperative planning, patient-specific instrumentation, teaching and learning.

## 3. Results

A total of 1648 results were obtained during the PubMed search. Of them, 62 articles met the inclusion criteria and were selected for the purposes of this review.

The current applications of 3D printing in hip orthopedic and trauma surgery are described below and summarized in Table 1.

### 3.1. Proximal Femoral Osteotomies

#### 3.1.1. Surgical Planning of Proximal Femoral Osteotomy in Developmental Hip Dysplasia [22]

The 3D-printed model is used to measure the femoral neck anteversion angle. The simulation of the proximal femoral osteotomy is performed with the contralateral side as a reference to restore the correct angles. Once the in vitro osteotomy is completed, the most suitable plate for fixation is chosen. It has been demonstrated that 3D images are more reliable than 2D CT scans when quantifying femoral anteversion in cases of hip dislocation in developmental dysplasia [23].

#### 3.1.2. Surgical Planning of Triplanar Osteotomy in Slipped Capital Femoral Epiphysis Sequelae [24]

A simulation of the surgical intervention is performed on biomodels by the surgeon. Based on the preoperative CT scan and clinical judgment, the osteotomy is carried out, and a wedge is removed from the trochanteric region to allow for a correction in flexion and valgus. The 3D model enables the surgeon to visualize the head–neck junction and optimize the physis orientation to achieve the desired correction. If the initial wedge is considered inadequate, additional cuts may be made to achieve an acceptable correction. Once the desired reduction is achieved, the fragments are secured with a Kirschner wire.

A similar technique has been employed for combined proximal and diaphyseal osteotomies in cases of sequelae of osteomyelitis. In this scenario, both lower limbs are printed, and the mirror image of the healthy femur is used as a reference to guide the osteotomy [25].

#### 3.1.3. Patient-Specific Instrumentation in Developmental Hip Dysplasia and Legg–Calvé–Perthes Disease [26,27,28]

Based on preoperative analyses, and in comparison with the contralateral side, varus and rotation angles, as well as the required length to shorten, are calculated. The guide includes two proximal sleeves for K wires which will help position the synthesis plate and guide the achievement of the desired varus. Two distal sleeves will assist in the fulfillment of the rotation. A proximal and distal saw guide permits the shortening. In order to succeed in the exact adaptation of the guide on the bone cortex, the authors suggest a Boolean operation, subtracting the femur from the guide.

Once the osteotomy is performed, the resulting bone fragment and the guide are removed, and the previously inserted wires serve as levers to achieve the correct positioning and orientation. The wires are then removed, and their holes can be used for inserting screws into the planned plate.

#### 3.1.4. Patient-Specific Instrumentation for Shepherd’s Crook Deformity (Pauwell’s Osteotomy) [29,30]

Preoperative planning is based on the Hilgenreiner epiphyseal angle, which has a normal value of 16 degrees. The difference between the patient’s angle and the standard value will determine the angulation of the fragment (the wedge) to be removed in the osteotomy. After defining this value, the surgical guide is designed.

The boundaries of the wedge are positioned proximally along a horizontal line that extends below the greater trochanter to the inferomedial femoral neck cartilage and distally by an oblique line from the lateral cortex to the first pin.

For the guide design, the lateral femoral surface is used, which is extracted and extruded using processing software for a perfect fit. The guide includes holes for Kirschner wires that secure the guide to the bone and channels for saw insertion.

#### 3.1.5. Patient-Specific Instrumentation for Acquired Complex Deformities of the Proximal Femur [31]

A better understanding of the deformity and the required correction plates is achieved through a mirror image of the contralateral bone, which is superimposed onto the affected femur. Once the desired correction is determined, a custom surgical guide is designed. This guide incorporates the individual features of the bone to ensure a precise fit in a specific location. Since a subvastus approach is commonly used, the intertrochanteric crest, along with the circumference of the femoral diaphysis, is often used as a reference point. This positioning can be supplemented by integrating stabilizing arms into the guide that can attach to other regions of the femur.

Once the guide is placed on the femur, reference pins are used, which are inserted into the bone through specific sleeves or chimneys. These pins serve as references for placing the remaining surgical guides. To prevent bone weakening, these pins may later be used as holes for plate screws.

In most cases, a cutting guide is designed for an oscillating saw, including references for inclination, direction, and depth. If curved osteotomies are required, a guide with multiple chimneys for drilling holes that can be connected later may be designed.

### 3.2. Periacetabular Osteotomy in Developmental Hip Displasia

#### 3.2.1. Surgical Planning [32,33,34,35]

After the acquisition of images with a CT scan, a 1:1 scale pelvic biomodel is printed on plastics such as ABS or PLA (or salt [34]) in order to perform an in vitro surgery on it. The fragment is rotated to achieve the position with the greatest coverage and stability of the hip, securing it with Kirschner wires. Once the simulation is performed, the model can be taken to the operating room to guide the surgeons in the in vivo procedure. The model can also be used to improve doctor–patient communication.

#### 3.2.2. Patient-Specific Instrumentation [36,37]

In the first step, the surgeon conducts virtual planning and design of the osteotomy according to the principles described by Ganz [38] or Tönnis. The design of the surgical guide is based on the surface of the resulting independent fragment after the osteotomy at the level of the quadrilateral plate. Two or three holes, 2 mm in diameter, are added for fixation using Kirschner wires. Another surgical guide can be designed to help with the rotation of the fragment. After virtually reducing the osteotomized fragment, it is possible to design a guide that would occupy the empty space between the pelvis and the free fragment, which can be used in surgery to guide the rotation and position the fragment. The available evidence suggests that patient-specific cutting guides have shown an increased precision while reducing surgical times and the need for intraoperative radiation compared to traditional methods [39].

A procedure with PSI has been described in adults for traumatic hip dysplasia, creating supraacetabular osteotomy guides in which the supraacetabular osteotomy is performed with a saw guide, and the retro acetabular osteotomy is carried out with the aid of positioning Kirschner wires [40,41].

### 3.3. Femoral Head Reduction Osteotomy in Avascular Necrosis of the Hip

#### Surgical Planning [42]

During the segmentation process, a 3D reconstruction of the femoral head is obtained. In a first step, a virtual planning of the osteotomy is performed on the computer, adjusting the osteotomy site to create the more spherical shape as possible. After executing the procedure on the processing software, the model is printed in order to assess the roundness of the femoral head and its congruity with the acetabulum.

In vitro planification surgery can also be performed if the femoral head 3D model is printed prior to virtual surgery.

A similar procedure has been described in cases of developmental hip dysplasia [35].

### 3.4. Primary Total Hip Arthroplasty

#### 3.4.1. Surgical Planning in Acetabulum Fracture Sequelae [43]

Once the affected hemipelvis is printed, the next step involves selecting the appropriately sized implant (acetabulum). This is done using the test components included in the set distributed by the commercial company. Additionally, the necessary augmentations are chosen to fill the existing cavities. Once the surgery has been planned in vitro, the biomodel is sterilized to make it suitable for use during the surgical intervention.

#### 3.4.2. Surgical Planning in Dysplastic Acetabulum [44,45]

The affected hemipelvis is segmented and printed in order to perform an in vitro surgery. In the first step, all the osteophytes around the Harris fossa are removed. Once the acetabular center is assessed, the reaming process is performed until the best fit is observed. Once the acetabular cup is positioned, the remaining defect is filled and measured with bone wax.

#### 3.4.3. Reaming of the Acetabular Component: The Positioning Ring PSI Method [46]

Originally described for developmental hip dysplasia sequelae, this method is based in the positioning of a ring guided by PSI, facilitating the subsequent reaming process, which is performed thanks to the orientation driven by the ring. For the PSI design, the contralateral (healthy) acetabulum serves as reference to address the true center of rotation. The angulation and anteversion of the cup are chosen according to the contralateral acetabulum, and its size is adjusted to avoid the disruption of the anterior or posterior walls of the true acetabulum. The ring represents the final positioning of the cup and is designed 2 mm wider than the expected cup size. The superior part of the original acetabulum is chosen as the reference landmark to position the PSI, which is fixed with K wires that are also oriented to assist in the reaming. The ring is latterly attached to the PSI. The reamer is kept in the middle of the ring, and the reaming process finishes when a reamer 2 mm below the size of the ring is used.

#### 3.4.4. Reaming of the Acetabular Component: The K Wire Crown PSI Method [47,48,49]

As in the previous case, a specular image of the healthy acetabulum is used in cases of dysplasia to localize the true center of rotation. The guide fits onto the bony surface of the acetabular rim or within the acetabulum, avoiding contact with the degenerative residual cartilage. The guide is created with multiple holes to place a crown of K wires around the future acetabulum, serving as a guide for the reaming. Once the reaming is finished, these wires also serve as a guide for the positioning of the cup.

#### 3.4.5. Reaming of the Acetabular Component: The Single K Wire in the Center of the Acetabulum PSI Method [49,50]

To establish the center of rotation of the acetabulum, it is visualized as part of a sphere. Spheres of different diameters are positioned in the acetabulum. The one with the maximum contact surface is chosen. Reference values of 40 degrees of inclination and 15 degrees of anteversion are used to draw a line from the center of the sphere to the acetabulum. The point where it intersects the acetabulum becomes our reaming center in which a guide will position a K wire. The positioning guide consists of the following components: a central hole for the wire and two or three branches that attach to the acetabular rim. After positioning the guide, a Kirschner needle is inserted through the guide. Reamers with a cannulated handle are required to allow the use of the guiding Kirschner wire.

#### 3.4.6. Patient-Specific Instrumentation for Femoral Neck Osteotomy [49,51]

Following the hip segmentation, the guide for the femoral neck osteotomy (above the tip of the lesser trochanter and at an angle of 45 degrees to the femoral shaft) is determined based on preoperative three-dimensional planning, including coronal (XZ) and sagittal (YZ) alignment of the femoral component.

Two different designs have been reported. One of the designs features a slot for performing femoral neck osteotomy using a saw, whereas the other design defines a blunt cutting surface at the edge of the guide. Both guides include a fixation area on the proximal part of the femoral neck.

It should be noted that these guides direct the osteotomy but cannot adjust the anteversion of the femoral stem.

#### 3.4.7. Patient-Specific Instrumentation for Femoral Diaphyseal Osteotomy in Crowe IV Developmental Hip Dysplasia [52]

This technique is useful when there is a greater length in the affected femur than in the contralateral side. A measurement is performed on both femurs to assess the length to shorten. The design of the surgical guide uses the surface of the posterior femur, distal to the lesser trochanter, ensuring adaptation to this anatomical region. The guide locks to the femur with K wires and limits the surfaces in which the two Z-shaped osteotomies should be performed in order to shorten the femur.

### 3.5. Revision Total Hip Arthroplasty

#### 3.5.1. Surgical Planning [53,54,55,56,57,58]

The pelvic bones, prosthesis and femur should be segmented and printed independently. These models can be reamed, and acetabular components can be placed in situ to assess the need for augmentations. In cases with significant defects (pelvic discontinuities), the placement of union supports using processing software may be necessary to prevent their movement during maneuvers. This technology enhances both sensitivity and precision in defect evaluation, providing better localization and increased efficiency compared to plain radiographs and CT scans [59].

#### 3.5.2. Training of Orthopedic Surgery Residents [10,45]

Anatomical biomodels are prepared for patients with or without acetabular deformities and from whom the femur has been removed. This process is undertaken for the assessment of the clinical case and the practical training of surgical interventions in resident training courses. In those cases with big defects, such as revision surgeries, models are printed in two colors to facilitate a better assessment of the defects.

### 3.6. Femoroacetabular Impingement

#### 3.6.1. Surgical Planning of Osteoplasty in Femoroacetabular Impingement [60]

The acetabulum the femoral head and neck models are printed separately. It has been demonstrated that the use of models contributes to modify the surgical approach regarding the location and amount of resection in 90% of femoral resection cases and 100% of acetabular resection cases.

#### 3.6.2. Patient-Specific Instrumentation for CAM Osteoplasty [61]

After acquisition and segmentation, two guides are created during processing. For the “Femoral Head Guide”, a sphere is designed on the healthy side, adapting its dimensions to those of the femoral head. Once this sphere is obtained, another sphere is created with a diameter 5 mm larger. Using a Boolean subtraction operation, the “custom sphere” is subtracted from the larger one, resulting in a hollow sphere with a wall thickness of 5 mm, which is cut to obtain a quadrant corresponding to a quarter of the total surface. A cylinder is added to be used as a handle for the guide during surgery. Another small cylinder is added to the front of the guide as a reference for the anterior and posterior parts.

A “Cervicocephalic Junction Guide” is created as a contoured plate, 6 mm in width and 3 mm in thickness, designed to fit the contour of the femur at the cervicocephalic junction. A cylinder is added as a handle. This guide will be used as a limit, marking the end of the femoral head.

#### 3.6.3. Self-Manufacturing of an Arthroscopy Simulator [62]

All unnecessary bone structures are removed during segmentation, retaining only the anterior superior iliac spine, the acetabulum, and the proximal femur. Since CT scans are performed in the supine position, the femur is repositioned to simulate traction conditions. This allows access to the central compartment for the simulated operations.

The simulator consists of two main parts: a soft component to simulate soft tissues and a hard component to simulate bone structures. As the acetabular labrum cannot be clearly defined in the CT scan, it is manually designed with a thickness of 3–4 mm and a width of approximately 8 mm. Nine fixed markers are incorporated on the surface of the acetabulum from the 8 o’clock to the 4 o’clock positions to facilitate the intra-articular identification of anatomical structures.

### 3.7. Osteosynthesis of Intracapsular Neck of Femur Fractures (Garden I or II)

#### Patient-Specific Instrumentation [63]

The virtual planning of the desired screw positions is performed during image processing. The design of a percutaneous surgical guide that sits perfectly on the bone cortex, taking into consideration the skin surface, is carried out. The guide includes three chimneys to position the Kirschner wires for the cannulated screws. To aid in the positioning of the guide, an extra chimney for a Kirschner wire that slides along the anterior surface of the femoral neck is included. This wire will help the surgeon maintain the guide in the desired position during the procedure.

### 3.8. Osteosynthesis of Extracapsular Neck of Femur Fractures

#### 3.8.1. Surgical Planning [64,65]

Care should be taken during segmentation in order to separate individual bone fragments. Using software, a virtual reduction of the fracture is performed, and the most suitable implant and positioning are selected. Printing is carried out twice for each fracture: as the unreduced, monoblock fracture, and subsequently, each of the individualized bone fragments is printed for manual reduction in a physical manner. The advantages highlighted by the authors for this surgical planning approach include reduced surgical time, decreased blood loss, and a shorter time until ambulation.

#### 3.8.2. Self-Manufacturing of Surgical Tools: A Device to Prevent Excessive Drill Penetration during Cortical Drilling of Distal Screws [6]

The design involves a screw nut with a side opening. This design allows the device to be inserted above the drill bit without requiring its removal from the drill. As the drill bit is introduced, the screw rotates around the nut. This has a dual purpose—on the one hand, it provides a secure grip for the surgeon to handle the system effectively, and on the other, it adjusts the length of the device to suit the specific requirements of each patient. This device has been shown to improve the precision of surgeons’ drilling, especially among those who are less experienced.

#### 3.8.3. Surgical Planning and Nail/Plate Pre-Bending in Atypical Femur Fractures with Bone Deformity [66,67]

For the selection of the most suitable nail, two orthogonal X rays of the femur are printed on paper. Various options for femoral nails and plates are placed on these paper prints to assess which one provides the best fit. Once the most suitable hardware is chosen, the complete femur is printed. An anatomical reduction of the fracture is manually performed if required, and the nail or plate is bent as needed. The standard antegrade nailing or MIPO technique is applied in vitro to assess the correct fit. In this simulation, all techniques that may be anticipated in real surgery, such as the use of Poller screws, can be employed.

#### 3.8.4. Surgical Planning and Plate Pre-Bending in a Peri-Implant Proximal Femur Fracture on an Arthrodesed Hip [68]

The model is printed including the pelvis and the entire femur. All artifacts caused by the DHS system used for the original arthrodesis are removed with a gouge during post-processing. Once these are removed, the desired implant is adapted. Various implants can be bent in order to choose the one with the best fit to the patient’s anatomy. The author’s preferred plate was a contralateral LISS plate in an anterograde direction. Temporary fixation is performed on the 3D biomodel using Kirschner wires to ensure that the screw trajectory will not penetrate the greater sciatic notch or the ilium. The definitive surgery is then carried out in a minimally invasive manner.

### 3.9. Trephination of Specific Trabeculae from Femoral Heads

#### Patient-Specific Instrumentation [69]

A CT scan with 0.625 mm slices is recommended for this purpose due to the size of the object of interest. According to the needs of the study, principal compressive trabeculae (PCT), principal tensile trabeculae (PTT) or bone tumors are localized using multiplanar reconstructions (MPR) with the segmentation software. Once the best corridor for the graft/biopsy extraction is selected, the design of the surgical guide is performed. Two cylinders (7.15 and 8.15 mm radii) are digitally positioned inside the main trabecular bundle, defining the channel for the trephine. This channel is created through a Boolean subtraction operation, creating a chimney. In the next step, a hemispherical dome is generated over the head. The head is then extracted with a Boolean subtraction operation, achieving a perfectly fit mold of the femoral head. Both the chimney and the dome are connected by a Boolean addition operation. The trabeculae are extracted with a trephine.

### 3.10. Soft Tissue Sarcomas of the Thigh

#### Surgical Planning and Teaching [70]

The segmentation is carried out by removing the muscles of the thigh and adjusting the transparency of the tumor. Bone, arteries, veins and nerves must be segmented separately applying masks of different colors.

**Table 1:** Current applications of in-house 3D printing in hip orthopedic surgery.

Category	Application	Type of Study	N*	Country
**Preoperative planning**	Proximal femoral osteotomy in DDH [22]	Retr. comparative	40 (20)	China
	Triplanar osteotomy in slipped capital femoral epiphysis sequelae [24]	Prosp. comparative	15 (5)	USA
	Periacetabular osteotomy in DDH [32,33,34,35]	Case report [32]	1 (1)	USA
		Case series [33]	42 (42)	USA
		Case report [34]	1 (1)	Japan
		Case series [35]	4 (4)	Italy
	Femoral head reduction osteotomy in AVN of the hip [42]	Case series	2 (2)	Turkey
	Primary THA in acetabulum fractures sequelae [43]	Case report	1 (1)	Spain
	Primary THA in dysplastic acetabulum [44,45]	Case series [44]	17 (17)	China
		Case series [45]	14 (14)	China
	rTHA [53,54,55,56,57,58]	Case series [53]	3 (3)	Ireland
		Case series [54]	17 (17)	Russia
		Case report [55]	1 (1)	Bulgaria
		Case report [56]	1 (1)	USA
		Retr. comparative [57]	45 (21)	Spain
		Retr. comparative [58]	72 (20)	Italy
	Osteoplasty in femoroacetabular impingement [60]	Case series	10 (10)	USA
	Osteosynthesis of extracapsular neck of femur fractures [64,65]	Prosp. comparative [64]	39 (19)	China
		Meta-analysis [65]	346 (172)	China
	Atypical femur fractures with bone deformity [66,67]	Case report [66]	1 (1)	South Korea
		Case series [67]	2 (2)	South Korea
	Peri-implant proximal femur fracture on an arthrodesed hip [68]	Case report	1 (1)	China
	Soft tissue sarcomas of the thigh [70]	Case series	2 (2)	China
**Patient-Specific Instrumentation**	Proximal femoral osteotomies in DDH and Perthes disease [26,27,28]	Prosp. comparative [26]	25 (12)	China
		Case series [27]	11 (11)	China
		Retr. comparative [28]	36 (16)	China
	Proximal femoral osteotomies in Shepherd’s Crook deformity [29,30]	Device presentation [29]		Italy
		Case series [30]	10 (10)	China
	Osteotomies for complex deformities of the proximal femur [31]	Device presentation [31]		Switzerland
	Periacetabular osteotomy in DDH [36,37]	Prosp. R. trial [36]	20 (8)	China
		Retr. comparative [37]	38 (20)	China
	Reaming of the acetabular component in THA [46,47,48,49,50]	Prosp. R. trial [46]	25 (12)	China
		Prosp. R. trial [47]	36 (18)	USA
		Case series [48]	24 (24)	Japan
		Review [49]		Japan
		Retr. comparative [50]	72 (40)	China
	Femoral neck osteotomy in THA [49,50,51]	Review [49]		Japan
		Retr. comparative [50]	72 (40)	China
		Case series [51]	30 (30)	Switzerland
	Femoral diaphyseal osteotomy in Crowe IV DDH [52]	Case series	12 (12)	China
	CAM osteoplasty [61]	Case report	1 (1)	India
	Osteosynthesis of intracapsular neck of femur fractures [63]	Prosp. comparative	40 (20)	China
	Trephination of specific trabeculae from femoral heads [69]	Prosp. R. trial	20 (10)	China
**Training**	Training of orthopedic residents in rTHA [10]	Case series	2 (2)	Brazil
	Hip arthroscopy simulator [62]	Cross sectional	19	China
	Soft tissue sarcomas of the thigh [70]	Case series	2 (2)	China
**Surgical tools**	A device to prevent excessive drill penetration during cortical drilling of distal screws	Prosp. R. trial	40	Spain

DDH (developmental dysplasia of the hip), AVN (avascular necrosis), THA (total hip arthroplasty, rTHA (revision total hip arthroplasty), N*: sample of patients. In brackets, patients in which 3D printing was used, Retr. (Retrospective), Prosp. (Prospective), R. (Randomized).

## 4. Discussion

We present a comprehensive reference guide with all the current applications of 3D printing in hip orthopedic and trauma surgery. A total of 27 applications of in-house 3D printing have been described in hip orthopedic surgery. The primary focus of most publications has been the assessment of procedural feasibility; yet the need for more robust evidence remains a pressing concern. Most of the presented studies are of limited evidence and present case series. The lack of homogeneity makes it challenging to obtain specific recommendations. Among the limited evidence available, specific applications have demonstrated promising outcomes. A reduction in the surgical time [24,26,27,28,35,37,57,58,63] can lead to a better efficiency of operating rooms as well as a reduction in the risk of infection. Additionally, a decreased fluoroscopy time [24,26,27,28,35,37] has been reported, pointing towards a potential reduction in radiation exposure for both patients and surgical teams. Surgical accuracy and precision [26,50,59] as well as safety [22,34] contribute to a more secure surgical environment. Three-dimensional printing technology has also led to increased patient satisfaction [57], as well as reduced bleeding [27,28,35,63] and overall costs [57,58]. Therefore, future prospective studies could provide more substantial evidence regarding the advantages of the described techniques. Nevertheless, it is crucial to acknowledge certain drawbacks associated with this technology. Some authors have pointed out that the time invested in three-dimensional planning and the design of surgical guides may not necessarily justify the time saved during the surgical intervention [71]. The 3D printing workflow presented in Appendix A includes different stages in which the technical work can be performed by a biomedical engineer. Currently, many hospitals worldwide lack the presence of a biomedical engineer who collaborates with orthopedic surgeons in performing these tasks, leading to a potential loss of resources. We firmly believe that in the future, many hospitals will incorporate technical personnel, enabling orthopedic surgeons to dedicate a significant portion of their time to surgical interventions, extending the indications of 3D printing in the common clinical setting. In an ideal workflow, the surgeon will request the 3D navigation tool, and all the acquisition, segmentation, design and printing will be performed automatically.

Two applications were described as self-manufactured surgical tools, such as the device to prevent excessive drill penetration during cortical drilling of the distal screw, which proved itself accessible, cheap and effective. Its benefits did not prove applicable only to novice surgeons but also to senior surgeons, improving their precision. The reliability shown by the hip arthroscopy simulator will permit orthopedic surgeons to train and improve their hip arthroscopy skills.

The strength of this work relies on the knowledge that it is the first publication detailing a comprehensive narrative review of all current applications of in-house 3D printing in hip orthopedic surgery in PubMed-Medline, which is a formidable search engine renowned for its reliability in healthcare literature. Nevertheless, it is essential to acknowledge that relying solely on a single database represents a limitation, a facet readers should bear in mind when interpreting our descriptive analysis.

## 5. Conclusions

There are many surgical applications of 3D printing in hip surgery, most of them based on CT images. Most of the publications lack evidence, and further randomized studies should be encouraged to assess the advantages of these indications.

## Appendix A. The 3D Printing Workflow

The processes carried out within medical 3D printing can be systematized into four phases:

- Image Acquisition: A dCT scan is at the present time the gold standard for image acquisition in orthopedics. We recommend 64-slice CT scans with a comfortable patient position, a slice thickness equal or less than 1.5 mm, an image matrix of 512 × 512 and a pitch equal to or less than 1. A reconstruction interval of 25–50% and a field of view (FOV) as closely tailored to the region of interest as possible should also be used [72,73]. Soft tissue kernel filters should be employed.
- Segmentation and Mesh Generation: This involves selecting the anatomical parts that you want to reconstruct in a 3D model. This process can be carried out using commercial software such as Materialise Mimics (Materialise NV, Leuven, Belgium) or open-source software like Horos (Horos Project, Annapolis, MD, USA), Invesalius (Centro de Tecnologia da Informacão Renato Archer, Campinas, SP, Brazil) or 3D-Slicer (BWH, Cambridge, MA, USA) [74]. In general, these programs allow the isolation of the anatomical structures of interest, taking advantage of the different radiological densities [75]. The next step is to extract the surface from the volumetric data, transforming the voxels into a polygonal model, a mesh composed of triangles and saved as an STL file (standard triangle language or standard tessellation language).
- Mesh Processing: In most cases, the triangular mesh requires correction or processing to eliminate errors and artifacts. It is crucial to compare the STL model obtained with the actual patient anatomy to ensure that corrections do not distort reality. This includes mesh correction, closing the ends of the model, optimizing internal structures, smoothing out artifacts and making model modifications [74].

3D Printing: In addition to printing, this phase of the workflow involves preparing and converting the segmented 3D model into a language that printers can understand for instructions. To accomplish this, the model needs to be imported into a software that will slice it into the various layers that will be used by the printer for fabrication. There are seven types of printers, but two of them are the most commonly found in hospital set ups: fused deposition modeling (FDM) printers and stereolithographic (SLA) printers. The former uses a wide range of plastics, which are fused at high temperatures and extruded through a nozzle, whereas the later uses liquid resin which is photopolymerized when it is exposed to light (curing).

## Appendix B. PRISMA Checklist

**Section and Topic**	**Item #**	**Checklist Item**	**Location Where Item Is Reported**
TITLE			
Title	1	Identify the report as a systematic review.	1
ABSTRACT			
Abstract	2	See the PRISMA 2020 for Abstracts checklist.	1
INTRODUCTION			
Rationale	3	Describe the rationale for the review in the context of existing knowledge.	1
Objectives	4	Provide an explicit statement of the objective(s) or question(s) the review addresses.	2
METHODS			
Eligibility criteria	5	Specify the inclusion and exclusion criteria for the review and how studies were grouped for the syntheses.	3
Information sources	6	Specify all databases, registers, websites, organizations, reference lists and other sources searched or consulted to identify studies. Specify the date when each source was last searched or consulted.	2
Search strategy	7	Present the full search strategies for all databases, registers and websites, including any filters and limits used.	2
Selection process	8	Specify the methods used to decide whether a study met the inclusion criteria of the review, including how many reviewers screened each record and each report retrieved, whether they worked independently and, if applicable, details of automation tools used in the process.	3
Data collection process	9	Specify the methods used to collect data from reports, including how many reviewers collected data from each report, whether they worked independently, any processes for obtaining or confirming data from study investigators and, if applicable, details of automation tools used in the process.	3
Data items	10a	List and define all outcomes for which data were sought. Specify whether all results that were compatible with each outcome domain in each study were sought (e.g., for all measures, time points, analyses) and, if not, the methods used to decide which results to collect.	3
	10b	List and define all other variables for which data were sought (e.g., participant and intervention characteristics, funding sources). Describe any assumptions made about any missing or unclear information.	Not applicable
Study risk of bias assessment	11	Specify the methods used to assess the risk of bias in the included studies, including details of the tool(s) used, how many reviewers assessed each study and whether they worked independently and, if applicable, details of automation tools used in the process.	3
Effect measures	12	Specify for each outcome the effect measure(s) (e.g., risk ratio, mean difference) used in the synthesis or presentation of results.	Not applicable
Synthesis methods	13a	Describe the processes used to decide which studies were eligible for each synthesis (e.g., tabulating the study intervention characteristics and comparing against the planned groups for each synthesis (item #5)).	3
	13b	Describe any methods required to prepare the data for presentation or synthesis, such as handling of missing summary statistics, or data conversions.	3
	13c	Describe any methods used to tabulate or visually display the results of individual studies and syntheses.	3
	13d	Describe any methods used to synthesize results and provide a rationale for the choice(s). If meta-analysis was performed, describe the model(s), method(s) to identify the presence and extent of statistical heterogeneity and software package(s) used.	3
	13e	Describe any methods used to explore possible causes of heterogeneity among study results (e.g., subgroup analysis, meta-regression).	Not applicable
	13f	Describe any sensitivity analyses conducted to assess robustness of the synthesized results.	Not applicable
Reporting bias assessment	14	Describe any methods used to assess risk of bias due to missing results in a synthesis (arising from reporting biases).	3
Certainty assessment	15	Describe any methods used to assess certainty (or confidence) in the body of evidence for an outcome.	Not applicable
RESULTS			
Study selection	16a	Describe the results of the search and selection process, from the number of records identified in the search to the number of studies included in the review, ideally using a flow diagram.	4
	16b	Cite studies that might appear to meet the inclusion criteria, but which were excluded, and explain why they were excluded.	Not applicable
Study characteristics	17	Cite each included study and present its characteristics.	3–14
Risk of bias in studies	18	Present assessments of risk of bias for each included study.	Not applicable
Results of individual studies	19	For all outcomes, present, for each study: (a) summary statistics for each group (where appropriate) and (b) an effect estimate and its precision (e.g., confidence/credible interval), ideally using structured tables or plots.	Not applicable
Results of syntheses	20a	For each synthesis, briefly summarize the characteristics and risk of bias among contributing studies.	4–9
	20b	Present results of all statistical syntheses conducted. If meta-analysis was done, present for each the summary estimate and its precision (e.g., confidence/credible interval) and measures of statistical heterogeneity. If comparing groups, describe the direction of the effect.	Not applicable
	20c	Present results of all investigations of possible causes of heterogeneity among study results.	Not applicable
	20d	Present results of all sensitivity analyses conducted to assess the robustness of the synthesized results.	Not applicable
Reporting biases	21	Present assessments of risk of bias due to missing results (arising from reporting biases) for each synthesis assessed.	9
Certainty of evidence	22	Present assessments of certainty (or confidence) in the body of evidence for each outcome assessed.	Not applicable
DISCUSSION			
Discussion	23a	Provide a general interpretation of the results in the context of other evidence.	9
	23b	Discuss any limitations of the evidence included in the review.	9
	23c	Discuss any limitations of the review processes used.	9
	23d	Discuss implications of the results for practice, policy, and future research.	9
OTHER INFORMATION			
Registration and protocol	24a	Provide registration information for the review, including register name and registration number, or state that the review was not registered.	9
	24b	Indicate where the review protocol can be accessed, or state that a protocol was not prepared.	9
	24c	Describe and explain any amendments to information provided at registration or in the protocol.	Not applicable
Support	25	Describe sources of financial or non-financial support for the review, and the role of the funders or sponsors in the review.	10
Competing interests	26	Declare any competing interests of review authors.	10
Availability of data, code and other materials	27	Report which of the following are publicly available and where they can be found: template data collection forms; data extracted from included studies; data used for all analyses; analytic code; any other materials used in the review.	10
